# Immobilization of the white-rot fungus *Anthracophyllum discolor* to degrade the herbicide atrazine

**DOI:** 10.1186/s13568-016-0275-z

**Published:** 2016-11-04

**Authors:** S. Elgueta, C. Santos, N. Lima, M. C. Diez

**Affiliations:** 1 Department of Environment and Sustainability, Instituto de Investigaciónes Agropecuarias, CRI La Platina, Av., 11610 Santa Rosa, Santiago Chile; 2 Department of Chemical Sciences and Natural Resources, University of La Frontera, Av. Francisco Salazar, 01145 Temuco, Chile; 3 CEB-Centre of Biological Engineering, Micoteca da Universidade do Minho, University of Minho, Campus Gualtar, 4710-057 Braga, Portugal; 4 Department of Chemical Engineering, University of La Frontera, Av. Francisco Salazar, 01145 Temuco, Chile

**Keywords:** Biopurification system, Herbicide degradation, White-rot fungus

## Abstract

Herbicides cause environmental concerns because they are toxic and accumulate in the environment, food products and water supplies. There is a need to develop safe, efficient and economical methods to remove them from the environment, often by biodegradation. Atrazine is such herbicide. White-rot fungi have the ability to degrade herbicides of potential utility. This study formulated a novel pelletized support to immobilize the white-rot fungus *Anthracophyllum discolor* to improve its capability to degrade the atrazine using a biopurification system (BS). Different proportions of sawdust, starch, corn meal and flaxseed were used to generate three pelletized supports (F1, F2 and F3). In addition, immobilization with coated and uncoated pelletized supports (CPS and UPS, respectively) was assessed. UPS-F1 was determined as the most effective system as it provided high level of manganese peroxidase activity and fungal viability. The half-life (t_1/2_) of atrazine decreased from 14 to 6 days for the control and inoculated samples respectively. Inoculation with immobilized *A. discolor* produced an increase in the fungal taxa assessed by DGGE and on phenoloxidase activity determined. The treatment improves atrazine degradation and reduces migration to surface and groundwater.

## Introduction

Atrazine is the most commonly used herbicide in Chile and perhaps in the world (Mesquini et al. 2015) and it is produced by the chemical giant Syngenta as a weed-killer. It is used for corn, sugarcane and sorghum, and reduces broadleaf and grassy weeds during pre- and post-emergence (Cabrera-Orozco et al. 2016). However, in the European Union, atrazine use was banned in 2004 due to persistent in groundwater. Exposure to atrazine can produce hermaphroditism in amphibians (Hayes et al. 2002). In addition, soil contamination by pesticides such as atrazine, during filling of sprayer tanks, can produce severe environmental impacts (Castillo et al. 2008; Grigg et al. 1997; Lozier et al. 2012).

Pesticides can be degraded naturally by microorganism and white-rot fungi (WRF) are used in biotechnological applications to undertake this biodegradation (Morgan et al. 1993; Castillo et al. 2000, 2008). WRF produce extracellular ligninolytic enzymes which degrade a wide range of other organic compounds (Rubilar et al. 2012). The most important role of WRF is in nature where the organisms recycling dead plant material which would otherwise accumulate in the environment making life on earth impossible. The ligninolytic enzymes from WRF are unique in that they can completely degrade lignin to carbon dioxide and water.

The ligninolytic enzymes include lignin peroxidases (LiP, EC 1.11.1.14), manganese peroxidases (MnP, EC 1.11.1.13) and laccase (Lcc, EC 1.10.3.2). These enzymes can be induced by lignocellulosic compounds or other organic compounds and their production is regulated by the availability of nutrients, temperature and inductors or inhibitors (Lorenzo et al. 2002; Rodríguez-Couto and Sanromán 2005; Baldrian 2008). The WRF *Anthracophyllum discolor* produces ligninolytic enzymes and mainly MnP in presence of pollutants such as chlorophenols, like pentachlorophenol (PCP), polycyclic aromatic hydrocarbons (PAHs) and synthetic dyes (Tortella et al. 2008; Elgueta and Diez 2010; Rubilar et al. 2011; Acevedo et al. 2011; Elgueta et al. 2012).

The biopurification system (BS) is an ecological and cost-effective technology to decrease pesticide contamination of soil and water (Castillo and Torstensson 2007). The BS is composed of straw, peat and soil and its efficiency is based on the ability to retain and degrade pesticides by indigenous soil microorganisms. Some reports on the importance of microbial communities involved in pesticide degradation in BS are available (Marinozzi et al. 2013). Studies have described the use of molecular methods such as denaturing gradient gel electrophoresis (DGGE) (Coppola et al. 2012; Marinozzi et al. 2013; Tortella et al. 2013). Coppola et al. (2012) described a change in microbial diversity after the addition of pesticides and demonstrated that yeasts and ascomycete filamentous fungi are involved in the pesticides degradation in BS. Tortella et al. (2013) evaluated the microbial community structure during atrazine degradation in a BS and observed little impact.

Agricultural and forestry residues generated as lignocellulosic wastes increase every year the environmental pollution. This leads to a loss of valuable natural compounds (cellulose, hemicelluloses and lignin) that can be converted to several value-added products (Rodríguez-Couto et al. 2001; Sanchez 2009). The biotransformation of lignocellulosic wastes can be attributed to microorganisms, especially the WRF, due their extracellular ligninolytic enzymes able to attack and transform not only lignin but also organic complex molecules as pollutants (Rao et al. 2014).

The majority of studies of atrazine degradation involve soil-based systems using bacteria (Newcombe and Crowley 1999; Fan and Song 2014; Zhang et al. 2014). However, some studies used WRF and Castillo et al. (2001) showed that *P. chrysosporium* in straw cultures was able to degrade 91% of the herbicide in 14 days of incubation. To take advantage of this performance, WRF can be immobilized in lignocellulosic supports increasing their ability to survive in the presence of indigenous soil microorganisms (Pepper et al. 2002). Sawdust has been proposed as an optimal support due to its capacity to support fungal metabolism (Walter et al. 2004; Smith et al. 2005). However, fungal immobilization has critical points that can affect the viability of fungi to survive in soil. Temperature and humidity can determinate the success of soil bioremediation using immobilized fungi (Walter et al. 2005; Schmidt et al. 2005; Ford et al. 2007).

Walter et al. (2004) found that wheat straw and a sawdust–cornmeal–starch-mix mixture (SCS) was a suitable carrier for *T. versicolor* for bioremediation of PCP in soil. Ford et al. (2007) evaluated PCP bioremediation by *T. versicolor* (3–175 g kg^−1^ inoculum) in highly contaminated field soils (100–2137 mg kg^−1^ PCP). They found that bioavailability and extractability of PCP in the contaminated soil may significantly increased after the bioaugmentation. In addition, Schmidt et al. (2005) found a strong correlation between the amount of fungal inoculum of *T. versicolor* used and fungal colonization in a soil bioaugmented for bioremediation. Rubilar et al. (2011) described the ability of immobilized *A. discolor* and *P. chrysosporium* on supports with wheat straw for soil bioremediation contaminated with PCP. These authors found high fungal growth rate and MnP production. The immobilization in wheat grains favoured the spread of fungi in the soil and consequently the pollutant degradation of more than 75%.

The main objective of the present study was to formulate a pelletized support to immobilize *A. discolor* and evaluate its capability to degrade the atrazine using a BS.

## Materials and Methods

Atrazine (99% pure), 3-methyl-2benzothiazolinone hydrazine (MBTH) and 3-dimethyl-amino benzoic (DMAB) were from Sigma-Aldrich (Santiago, Chile). All other chemicals and solvents were of analytical reagents grade and were purchased from Equilab Ltda. and Merck S.A. (Chile).

### Fungus

*Anthracophyllum discolor* Sp4 CCCT 16.5 (Colección Chilena de Microrganismos Tipo CCCT, WDCM 1111) was supplied by the Environmental Biotechnology Laboratory, Universidad de La Frontera, Chile. The fungus was transferred from malt extract agar (MEA, 30 g L^−1^ mal extract, 15 g L^−1^ pH 5.2, Sigma-Aldrich). Slant culture of glucose malt extract agar (GMEA, MEA plus 10 g L^−1^ glucose) plates and incubated at 25 ± 1 °C for seven days. The fungus was stored at 4 °C and regularly sub-cultured to maintain viability.

### Pelletized support

Corn meal (Protein 7.2%, Fat 0.4%, Crude Fiber 0.4%, Ash 0.36% and Carbohydrates 78%) and potato starch (pH 6–7.5, solubility 50 g L^−1^, and reducing matter (as maltose) ≤0.7%) were purchased from Merck S.A Chile (99% purity), flaxseed meal was purchased from ANASAC S.A. (Temuco, Chile). The sawdust from pine (moisture 10%, ash 0.7% and volatile 65%) was obtained from the forestall ARAUCO (Concepcion, Chile) mill and sieved through 5 mm. Lignosulphonate (Ca–Mg formulated) was purchased from Lignotech Iberica (Cantabria, Spain). These components were used to formulated the support F1 (starch 6%, corn meal 2% and flaxseed 15%), F2 (starch 8%, corn meal 5%, flaxseed 10%) and support F3 (starch 10%, corn meal 8%, flaxseed 5%). Lignosulphonate (3%) and sawdust (74%) were incorporated in all formulations. A pellet mill (ZLSP300B R-type, Shangai, China) was used to obtain a pelletized support (PS) of eight mm length and six mm diameter, with a 10% humidity of dry weight after the pelletization.

### *Anthracophyllum discolor* immobilization

The fungal immobilization was performed as described by, Walter et al. (2004) with some modifications. The mycelium scraped from GMEA plates was homogenized in a sterilized blender for 1 min with liquid Kirk medium (LKM), (10 g L^−1^ peptone, 2 g L^−1^ glucose, 2 g L^−1^ KH_2_O_4_, 0.5 g L^−1^ MgSO_4_, 0.1 g L^−1^ CaCl_2_, 2 g L^−1^ thiamine and 10 mL^−1^ mineral salts) (Tien and Kirk 1988). Five mL of this inoculum were transferred to 250 mL of LKM and incubated at 25 °C for seven days. This culture was used to prepare: (i) a coated pelletized support (CPS) formulated with 50 g of F1, F2 and F3. A suspension of 250 mL of mycelium from LKM and 100 mL alginate (2%) was shaken and homogenized for 1 min using a vortex mixer. PS were sprayed at 15 cm with this suspension, collected and allowed to harden for 3 min in 3% CaCl_2_; (ii) an uncoated pelletized support (UPS) was formulated by transferring 50 g of F1, F2 and F3 to plastic bags and adding 10 mL of GMEA only. These bags (UPS) were inoculated with five agar plugs with mycelium of *A. discolor* grown on GMEA at 25 °C for 30 days. All the treatments were in triplicate.

In order to evaluate the growth and ligninolytic enzymes activities of *A. discolor* in the various CPS and UPS treatments, 50 g of each F1, F2, and F3 formulation was incubated for 15 days at 25 °C. Fungal growth was determined daily measuring the fungal biomass produced per day. After this, the viability of *A. discolor* in CPS and UPS was monitored at 4 and 25 °C during 3 months: briefly, every 5 days from each bag, 2 pellets were taken from plastic bags and transferred to a plate with GMEA and PDA media to check the fungal capacity to grow. The fungal mycelium was monitored every day until complete the plate. At the same time, pellets without inoculation were checked in the same conditions. The pellets were analyzed by Scanning Electron Microscope (SEM). Then the optimal immobilized support obtained was selected to inoculate the BS on the basis of this information.

### Biological activity determinations

Ligninolytic enzymes were extracted from 1 g of each sample with 5 mL 50 mM sodium malonate (pH 4.5). The samples were centrifuged at 10,000 rpm and stored up to 24 h at 4 °C prior to analyses. All the determinations were in triplicates. The Lcc activity was determined with 2,6-dimethoxyphenol (DMP) as the substrate in sodium malonate (pH 4.5).

The MnP activity was determined by monitoring the oxidation of DMP spectrophotometrically at 30 °C. One enzymatic activity was defined as the amount of enzyme transforming 1 µmol DMP min^−1^ (Moreira et al. 1997). The manganese-independent peroxidase (MiP) was monitored at 468 nm and corrected by Lcc activity (De Jong et al. 1994). The lignin peroxidase (LiP) was monitored at 310 nm for 2 min. One enzymatic activity unit was defined as the amount of enzyme transforming 1 µmol of veratryl alcohol per minute (De Jong et al. 1994).

For all degradation assays, phenoloxidase activity (PO) was determined every seven days and performed using MBTH and DMAB (Castillo et al. 1994). The PO measurements represent the sum of MnP, Lip and Lcc (Castillo and Torstensson 2007). Briefly, biomixture of the BS samples (10 g) were shaken (150 rpm) for two hours with 25 mL 100 mM succinate-lactate buffer (pH 4.5) and then centrifuged at 4000 rpm for 20 min. The activity was monitored at 590 nm in a spectrophotometer. In order to obtain information about metabolic activity during atrazine degradation, respiration activity was measured as CO_2_ produced absorbed in a 0.2 M NaOH solution at 20 °C during the experiment. The values were expressed in mg CO_2_ g^−1^ dry biomixture. Experiments were conducted using three independents replicates, statistical analysis was undertaken using multi-way analysis of variance, and the averages were compared by Tukey range tests.

### Scanning electron microscopy

Photomicrographs were taken using SEM (Leica/Cambridge Instrument S360, Cambridge, UK). Samples with two cm length of the PS with immobilized fungus were cut using a sterilized knife and fixed with 2.5% glutaraldehyde for 1.5 h at 4 °C followed by 0.1 M cacodylate salt buffer pH 7 for 3 0 min, then post-fixed with 1% osmium tetroxide, dehydrated with acetone, dried and metalized with gold.

### Biomixture formulation

The BS was formulated by using Andisol top soil (Freire serie) mixed with wheat straw and peat in a volumetric proportion of 1:2:1 (w:w:w). The soil used was collected from 0–15 cm deep and sieved to 3 mm. The soil pH was 5.4 with 18 mg kg^−1^of available nitrogen, 17 mg kg^−1^ of available phosphorus and 12% of organic matter. The straw (pH 5.9, 0.5% N, 9% lignin and 66% organic matter) was collected from crop residues at a local farm and was chopped to obtain fractions of 2 cm length. Commercial peat was purchased from ANASAC S.A. (Temuco, Chile), contained 33% cellulose and 21% lignin. The biomixture formulated was stored in a polypropylene bag at 4 °C until use. The final biomixture provided pH 4.8, 0.5% total N and 30% organic carbon. The above analyses where performed by the accredited Soil Laboratory in Universidad de La Frontera, Chile. In order to obtain a homogenous biomixture, the formulation was vigorously shaken in a plastic bag of 5 L and the moistened with distilled water to 60% water holding capacity (WHC). The biomixture was incubated for 30 days at 20 °C in a polypropylene bag until their use. In order to establish a BS, biomixture (1 kg) was transferred to 5 L glass pot and inoculated with 10% (w/w) immobilized fungus (dry weight) on PS at depth of 5 cm below the surface of BS. Thereafter, 60 mg kg^−1^ atrazine (high concentration similar to a spillage) was spread over the BS.

Four BS were prepared: (a) biomixture + atrazine, (b) biomixture + fungal inoculum, (c) biomixture + atrazine + fungal inoculum and (d) biomixture as control. Each experiment was carried out in triplicate using a destructive sampling mode. To evaluate the microbial communities an analysis by DGGE was done and samples were extracted for DNA analysis after 0, 20 and 30 days of incubation. The time-course of PO activity, residual atrazine and respiration activity (CO_2_) were determined for 30 days.

### Analytical procedures

Atrazine was extracted with 20 mL of methanol from 10 g of soil. Incubation was performed for 1 h at 25 °C with shaking at 350 rpm (Diez et al. 2013). Samples were sonicated at full power for 30 min, centrifuged at 10,000 rpm for 10 min and filtered through 0.2 µm PTFS membrane filters (Merck, Santiago, Chile). For each sample, the procedure was performed in triplicate. The concentrations in the final supernatants were measured by high performance (HPLC, VWR Hitachi). Samples were injected using a Rheodyne 7725 injector. The HPLC had a Merck Hitachi L-7100 pump in a Merck Hitachi L7455 (Knauer, Germany) diode array detector set at 290 nm. The mobile phase was methanol (100%) and the flow rate was set a 1 mL min^−1^. The recovery rate of atrazine was greater than 85%. The half-life value of atrazine were obtained by using the first-order kinetic equation as Concentration = C0 e^−kt^, and from this equation, it was obtained: *T*_*1/2*_ = *Ln(2)/k*; where *k* is the first order-rate constant (d^−1^).

### Microbial community DNA analysis

The microbial community composition in soil was evaluated at 0, 20 and 30 d by DGGE using the specific primer set for bacteria and fungi described by Tortella et al. (2013). Briefly, soil DNA was extracted (0.5 g) using the FastDNA® Spin kit following the manufacturer’s instructions. The quantity and the quality of the DNA extracted was determined using 1% agarose gel electrophoresis in 0.5× Tris–borate-ethylenediaminetetraacetic acid (EDTA, TBE) buffer.

The composition of the bacterial communities in the biomixture was determined using PCR amplification of the 16S rRNA gene, a nested PCR approach, which was followed by the DGGE analysis. The PCR products obtained were nested with primers 341f + GC-534r, which provided a 200 bp product, which was analysed using PCR-DGGE. For the analysis of the fungal community, the DNA was amplified using primers ITS3-GC and ITS4, which generated a fragment of approximately 200 bp that was suitable for the PCR-DGGE analysis. All the PCRs were performed in 50 μL reaction volumes containing 10 µL buffer ‘Green GoTaq® Flexi’ 5× , 2.5 µL magnesium chloride (MgCl_2_, 25 mM), 1 µL of DNTP’S (10 mM), 1 µL of each primer (10 mM), 0.25 µL Taq (5 U µL^−1^, Go Taq® Flexi DNA polymerase, Promega Corp), 3 µL ADN (50 ng µL^−1^), and 31.25 µL water.

The PCR-DGGE analyses were performed using a Bio-Rad DCode system. The polyacrylamide gels (8%) in 1 × Tris–acetate-EDTA (TAE) buffer (40 mM Tris base, 20 mM acetic acid, and 1 mM disodium EDTA, pH 8.2) were prepared with a denaturating gradient of 40–70% for bacteria, and 30–60% for fungi (where 100% denaturant contained 7 M urea and 40% formamide). The electrophoresis was run for 17 h at 60 °C and 80 Volts. The images were captured using a digital camera, and all the DGGE gel pictures were analysed using the Phoretix 1D analysis software.

## Results

### *A. discolor* immobilization and ligninolytic enzyme activities

**Fig. 1:**
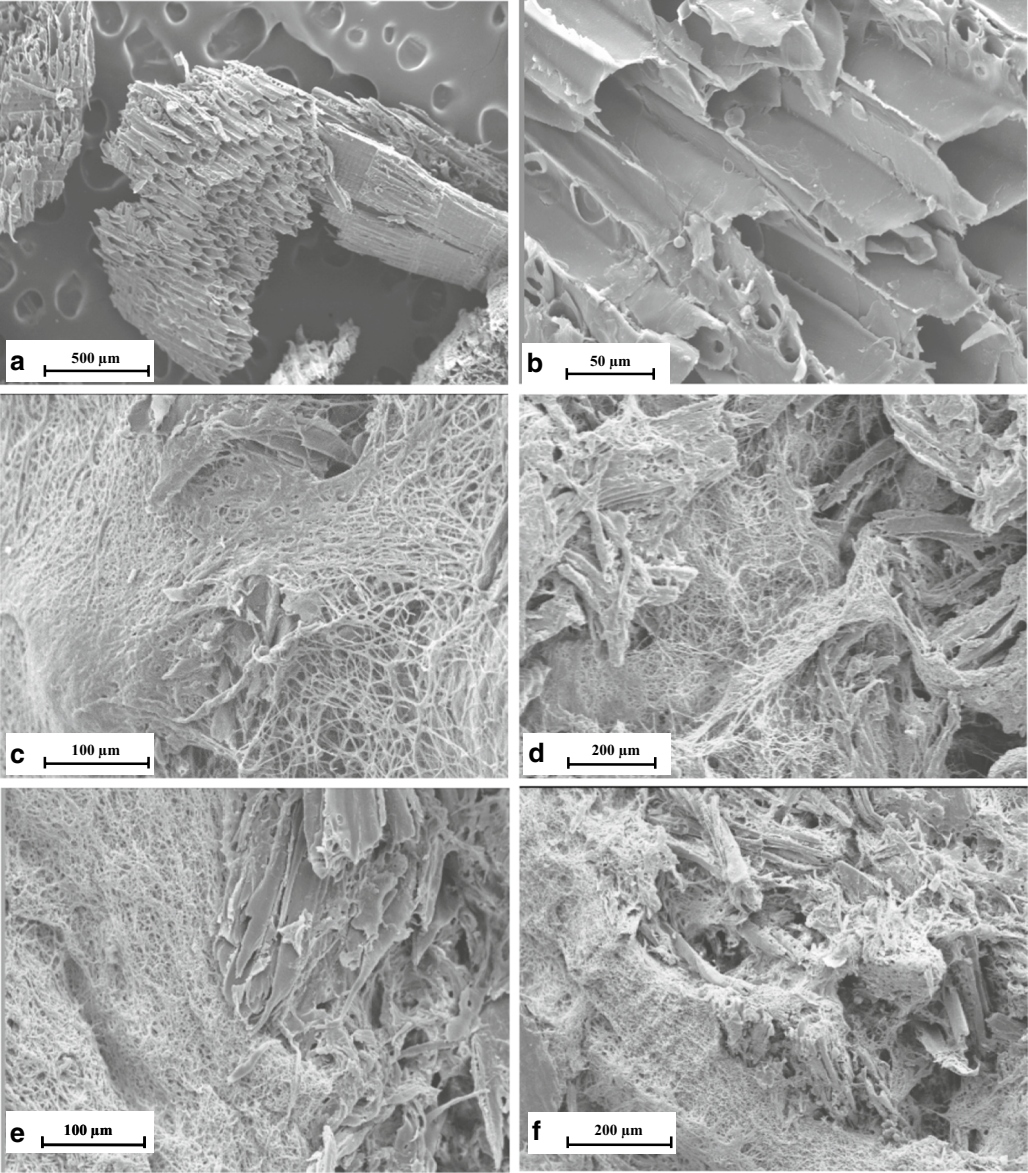
Microphotographs obtained by SEM for Anthracophyllum discolor on UPS-F1 at two temperatures after 30 days of incubation. mixture of pelletized supports (**a**) sawdust (**b**) Surface and core at 25 °C (**c**, **d**) Surface and core (**e**, **f**) at 25 °C

The fungus produced uniform growth across the surfaces and cores of the PSs during immobilization. However, the immobilization of *A. discolor* on UPS was faster than CPS during the overgrowth period (data not shown). Overall, uniform fungal growth across the interspaces of the PSs was observed. The growth of *A. discolor* in the surface and core of UPS-F1 at 4 and 25 °C can be observed by the SEM photomicrographs (Fig. 1c, d–f). The controls of pelletized supports and sawdust (Fig. 1a–b) indicated not evidence of fungal mycelium during the evaluation Similar growth was observed for CPS-F1, F2 and F3 and UPS-F2 and F3 (data not shown). UPS-F1 immobilization was chosen for further evaluations based on its capacity to permit the fungus to growth and produce high levels of MnP. The lignocellulosic matrixes after the inoculation not showed any microbial colonization during the storage, confirming that after the pelletizing process its where innocuous.

**Fig. 2:**
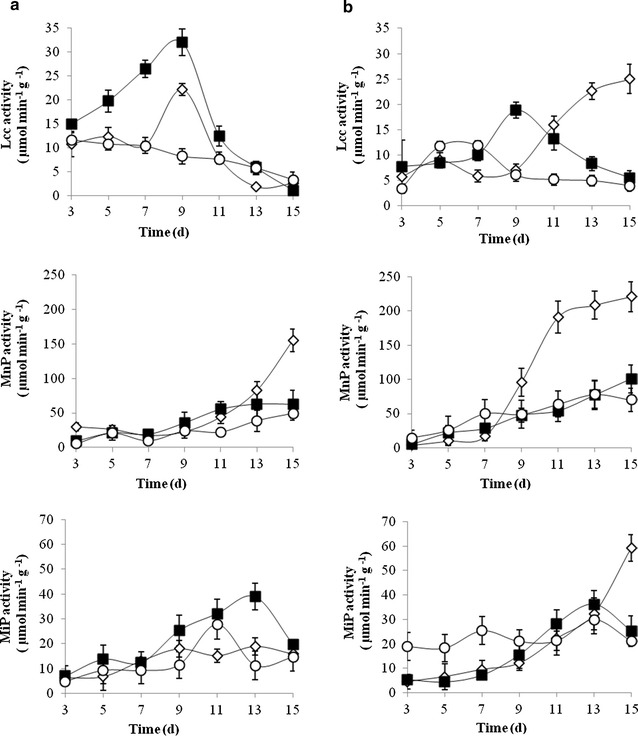
Time-course of ligninolytic enzyme activities (Lcc, MnP and MiP) produced by *Anthracophyllum discolor* in coated (**a**) and uncoated (**b**) pelletized supports for different formulations [F1 (*Lozenge*), F2 (*Black square*) and, F3 (*white circle*)], with incubation temperature at 25 °C

The time-course of Lcc, MnP and MiP produced by *A. discolor* in coated and uncoated pelletized supports for different formulations (F1, F2 and F3) was determined (Fig. 2). MnP showed the highest activity for both immobilizations. LiP was not detected during the assay. The ligninolytic enzyme activities in CPS (Fig. 2a) were evaluated for all formulations (F1, F2 and F3). In formulation F2, Lcc showed peak activity of 32 µmol min^−1^g^−1^ after 9 days. However, for F1 and F3 the activity decreased over time. The MnP was similar for all formulations, with a peak activity of 155 µmol min^−1^g^−1^ on day 15 for F1. The production of MiP was continuous and reached a peak activity of 38 µmol min^−1^g^−1^ on day 13. However, on day 15, the MiP activity decreased below 20 µmol min^−1^g^−1^.

Enzymes activities for UPS (Fig. 2b) were slightly higher than for CPS. The formulation F1 showed a peak Lcc of 25 µmol min^−1^g^−1^ at day 15. Formulation F1 showed the highest MnP activity on day 7, reaching the highest activity of 220 µmol min^−1^g^−1^on day 15. However, for F1 and F3 the activities were below 25 and 100 µmol min^−1^g^−1^ respectively. The MiP production was similar for all formulations with a peak activity of 36 µmol min^−1^g^−1^ for F1. However, on day 15, the activity decreased below 25 µmol min^−1^g^−1^.

### Atrazine degradation

The immobilization of *A. discolor* allowed hyphae to penetrate into the biomixture of the BS and the fungus was effective at accessing atrazine better that other non-immobilized microorganisms. However, a cell to atrazine intimate contact may not be required as the enzymes are extracellular. After the selection of UPS-F1, the atrazine degradation and fungal activities in a biomixture were evaluated.

**Fig. 3:**
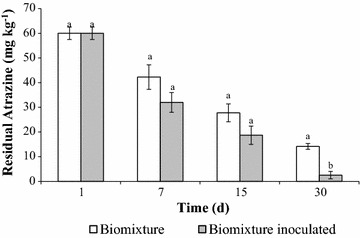
Residual atrazine (mg kg^−1^) during 30 days of incubation at 20 °C in a biomixture inoculated. *Different lowercase letters* indicate significant (P ≤ 0.05) differences between biomixtures across non- and inoculated conditions

Atrazine degradation was higher in the inoculated BS than in non-inoculated (Fig. 3). This result is relevant and novel due to the difference between atrazine degradation of 76% (non-inoculated) and 96% (inoculated) represent 12 mg kg^−1^ more of atrazine degraded in the system inoculated, a 20% of the total atrazine added to the system (60 mg kg^−1^). In general, in agricultural practices the normal field rate of atrazine is about 1 mg kg^−1^ used in corn production.

### Biological activities in the biomixture system

**Fig. 4:**
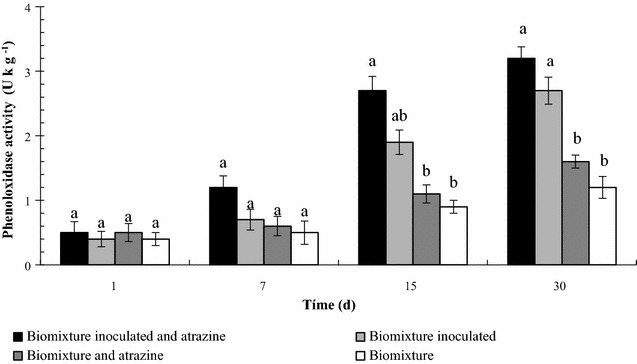
Phenoloxidase activity (U kg^−1^) after 30 days at 20 °C, in a biomixture inoculated. *Different lowercase letters* indicate significant (P ≤ 0.05) differences between biomixtures across different conditions

The non-inoculated BS gave phenoloxidase activities of 0.4–1.2 U kg^−1^. In contrast, the PO activities in the inoculated BS varied from 0.4–3.2 U kg^−1^. After 15 days of incubation the PO activity in the system inoculated were doubled, in respect to the non-inoculated BS. Overall, for inoculated and non-inoculated BS the enzymatic activity increased over time reaching its maximum on day 30 (Fig. 4).

**Fig. 5:**
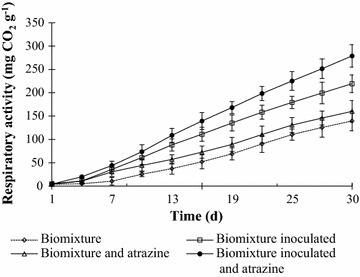
Cumulative respiration activity (mg CO2 g^−1^ biomixture) after 30 days at 20 °C, in a biomixture inoculated with immobilized *Anthracophyllum discolor*. Each value is the mean of three replicates, and the *error bars* show the standard deviation of the mean

A high respiration rate (279 mg CO_2_ g^−1^ d^−1^) was observed in the inoculated BS plus atrazine, followed by the levels for the simply inoculated BS (219 mg CO_2_ g^−1^ d^−1^). The high respiration rate of BS inoculated can be related with the high level of atrazine degradation, this can be corroborated by works of Castillo and Torstensson (2007). It can also be attributed to the presence of readily degradable carbon sources in that environment. By way of confirmation, minor respiration rates were observed for non-inoculated BS (139 mg CO_2_ g^−1^ d^−1^) (Fig. 5). Castillo and Torstensson (2007) observed that the presence of straw in biomixture increased the activity during the degradation of pesticides, containing readily available carbon sources that promotes the respiration activity. While polysaccharides are sources of energy for microorganisms in the soil, the degradation of lignin and humic substances does not provide enough energy to maintain decomposition.

### DGGE analysis in the biomixture

**Fig. 6:**
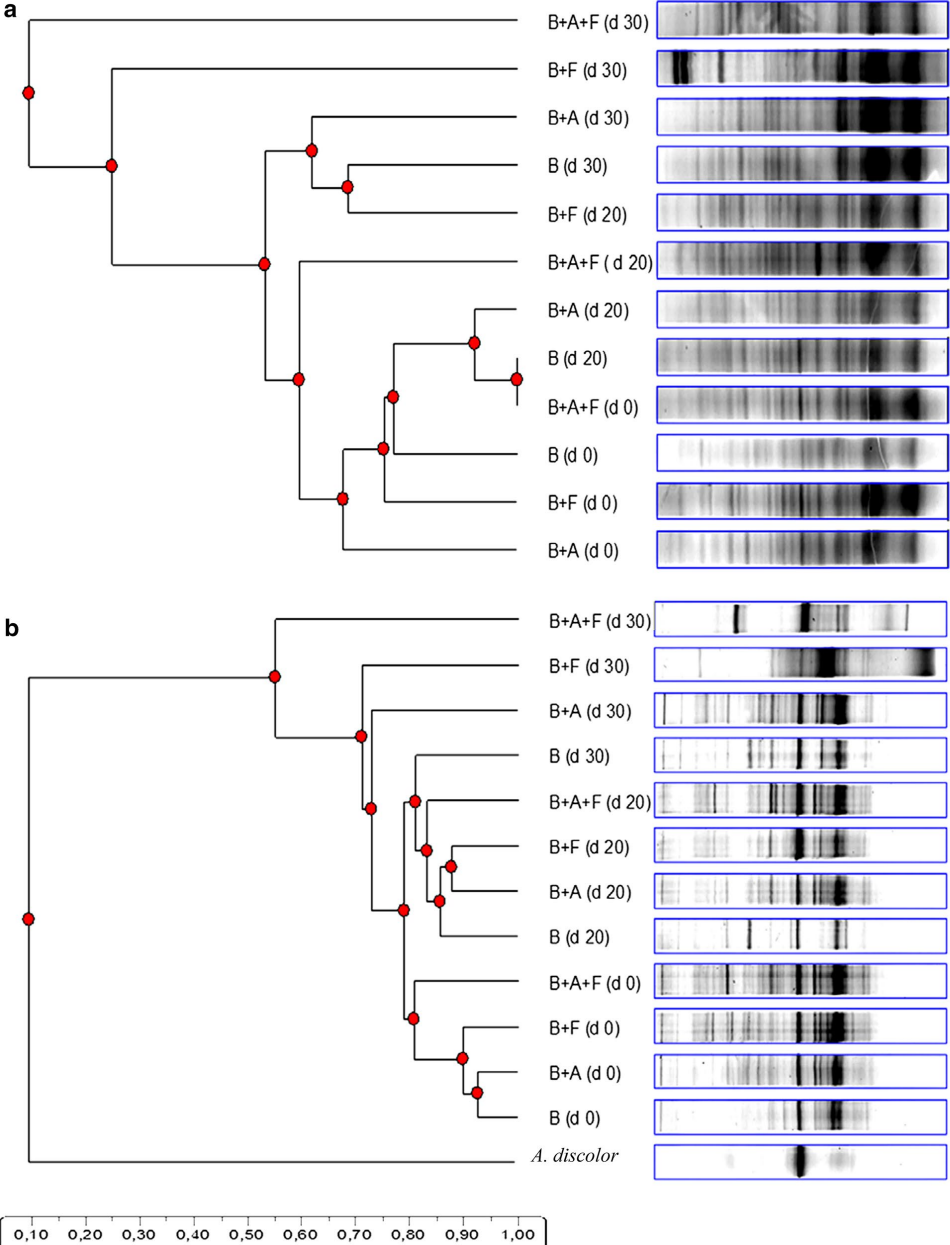
Microbial communities in the biomixture evaluated by DGGE analysis during 30 days, **a** Bacterial communities; **b** Fungal communities. The code used: *B* (Biomixture), *B* *+* *A* (biomixture and atrazine), *B* *+* *F* (biomixture inoculated), *B* *+* *A+F* (biomixture inoculated and atrazine)

The effect of *A. discolor* UPS-F1 in the biomixture contaminated with atrazine on bacterial and fungal communities was evaluated by DGGE analysis (Fig. 6). The patterns of bacterial communities obtained are shown in the Fig. 6a and had 65% similarity at the end of the analysis. The inoculated biomixture plus atrazine indicated a stimulation of bacteria during the evaluation. On day 20, a shift on patterns of bacterial communities is observed when compared with the patterns of bacterial communities obtained without atrazine.

The biomixtures showed fewer bands than the control on day zero which can be attribute to the atrazine effect. In addition, the results indicated that there was little change on day 30, mainly for the inoculated biomixture. This process can be related to the normal stabilization of native microbial communities in the inoculated biomixture.

## Discussion

The results demonstrated that the proliferation of fungal mycelia of *A. discolor* was significantly higher in the formulation F1 (high flaxseed content). Flaxseed contains approximately 28% lignocellulose, 23% lignin, 25% hemicellulose, and 47% cellulose (Coskuner and Karababa 2007). At least some of the cellulose present in flaxseed may be readily degraded by WRF encouraging good growth and increasing the amount of ligninolytic enzymes that can be produced (Sanchez 2009). The results obtained of MnP with high activity in F1 are concordant with those previously published which associate *A. discolor* with a high capacity to produce MnP (Tortella et al. 2008; Rubilar et al. 2011; Bustamante et al. 2011; Acevedo et al. 2011; Elgueta et al. 2012). Nevertheless, other fungi can produce different ligninolytc activities for bioremediation process. Walter et al. (2004) evaluated immobilized *T. versicolor* in lignocellulosic supports for soil bioremediation of pentachlorophenol, obtaining high levels of laccase of over 700 µmol min^−1^g^−1^ by day 19. Similar results were obtained by Pinto et al. (2016) exploring the potential of novel biomixtures inoculated with *Lentinula edodes* for degradation of selected pesticides in biobed systems. They found high proliferation of fungal mycelia in a cork substrate by SEM microscopy. Elgueta et al. (2016) evaluated the atrazine degradation in a bioaugmented biomixture with different immobilized white-rot fungi. They found high fungal growth by *Trametes versicolor* HL 01 *Stereum hirsutum* Ru 104 and *Inonotus* sp *SP2* analyzed by SEM micrographs immobilized in lignocellulosic supports.

In our study, the initial concentration of atrazine was 60 mg kg^−1^ similar to that which could be expected from an important on-farm spillage. The inoculated biomixture presented a half-life (t_1/2_) of 6 days whereas for the non-inoculated biomixture was 14 days, indicating a high degradation efficacy. The limitation in nitrogen content in peat biomixture and high C/N ratios enhance the degradation of pesticide mediated by white-rot fungi Rodríguez-Rodríguez et al. (2013). The present results of atrazine degradation are higher than those obtained in other systems: Bending et al. (2002) evaluated the capacity of nine species of white-rot fungi to degrade mono-aromatic pesticide in a biobed biomixture. They found that *Coriolus versicolor*, *Hipholoma fasciculare* and *Stereum hirsutum* degraded 51, 61 and 57% respectively of 20 mg kg^−1^ of atrazine after 42 days of incubation. In addition, Tortella et al. (2013) evaluated a biomixture supplemented with terpenes during atrazine degradation. These authors found that the biomixture suplemented with 50-µg kg^−1^ of limonene or eucalyptol gave t_1/2_ of atrazine of 9 days, which was significantly lower than the control (13 days). Ruiz-Hidalgo et al. (2014) used rice husk inoculated with *Trametes versicolor* to degrade a 55% of carbofuran in 34 days using a biomixture. In addition, Ruíz-Hidalgo et al. (2016) also evaluated the optimization of a bioaugmented biomixture for the degradation of carbofuran using *T. versicolor.* They found the use of rice husk as bioaugmentation agent supports the cost-effectiveness of the pesticide degradation in the system. In addition, they evaluated ecotoxicological effects from lixiviates from a bioaugmented biomixture with *T. versicolor* for carbofuran degradation suggesting that the optimized biomixture decrease the residual toxicity and the potential chronic effect on aquatic life. Madrigal-Zúñiga et al. (2016) found the bioaugmentation by *Trametes versicolor* improved the mineralization of carbofuran with a half-live of 3.4 and 8.1 in rice husk-based biomixture.

The presence of the inoculum in the BS increased the PO activity in the biomixture. This result can be related to the high content of lignin related compounds present in the biomixture that can be degraded by WRF. The biomixture contains carbon sources, like cellulose, linked to lignin degradation, which could explain why pesticide degradation is correlated with PO activity (Castillo et al. 2001). Results obtained in this present work are corroborated by previous studies published by, Karas et al. (2011) who described *T. versicolor* as an efficient degrader of pesticides in wastewaters from the fruit packaging industry. However, they found contrasting results for ligninolytic activities and no correlation with pesticide degradation was found.

For fungal communities the DGGE patterns in the biomixture samples had high (>70%) similarity indices (Fig. 6b). The inoculated biomixture plus atrazine showed stimulation for some fungal groups, mainly at the end of experiment. Moreover, the DNA of *A. discolor* was used as an indicator during the experiment due their presence over the time. The DGGE analysis showed differences of microbial community between inoculated and non-inoculated biomixture. The presence of *A. discolor* in all treatment over the time can be explained based on the similarities with indigenous basidiomycetes present in the biomixture and the high amount of readily metabolized carbon sources. The level of colonization of WRF can be modulated by reduction of pH in the biomixture, increasing the fungal activity and pesticide degradation (Rodríguez-Rodríguez et al. 2013).

Results obtained in this present work are corroborated by previous studies by, Tortella et al. (2013) who described the impact of bacterial communities in the same biomixture (soil, straw, peat) contaminated with atrazine. The inoculation of *A. discolor* in the biomixture produced changes in the structure of the microbial communities between inoculated biomixture and control. Tortella et al. (2013) found high similarity indices (>85%) in DGGE patterns of fungal communities and no difference between inoculated and non-inoculated biomixture. However, the authors observed differences in the DGGE patterns that were associated with the pesticide application. In addition, Coppola et al. (2012) showed an evident modification of microbial diversity in a biomixture contaminated with penconazole, dimethomorph, metalaxyl, azoxystrobin, cyprodinil and fludioxonil by DGGE analysis. The highest degradation of atrazine in the system inoculated, allow to confirm the presence of *A. discolor* in the process through the DNA showed in the DGGE analysis.

In conclusions, the inoculation of the biomixture with immobilized *A. discolor* increased atrazine degradation. The formulation F1 with the high proportion of flaxseed meal (15%) gave the highest MnP activity. The atrazine degradation was higher in the inoculated biomixture than in the non-inoculated one. The t_1/2_ of atrazine decreased from 14 to 6 days. The inoculation with immobilized *A. discolor* produced an increase in the abundance of fungal taxa as well as in the PO activity. Therefore, the main conclusion of this work is that the BS inoculated has 20% more of atrazine degraded that is an important reduction from the initial concentration 60 mg kg^−1^.

## Abbreviations

BS: biopurification system
F1, F2, F3: formulations
PS: pelletized support
CPS: coated pelletized supports
UPS: uncoated pelletized supports
SCS: sawdust–cornmeal–starch-mix mixture
WRF: white-rot fungi
Lcc: laccases
MnP: manganese peroxidases
MiP: manganese-independent peroxidase
LiP: lignin peroxidases
DGGE: denaturing gradient gel electrophoresis
GMEA: glucose malt extract agar
WHC: water holding capacity
SEM: scanning electron microscope
PO: phenoloxidase
MBTH: 3-methyl-2-benzothiazolinone hydrazone
DMAB: 3-dimethyl-amino benzoic acid
PCP: pentachlorophenol
DMP: 2,6-dimethoxyphenol
